# IL-27 Counteracts Neuropathic Pain Development Through Induction of IL-10

**DOI:** 10.3389/fimmu.2019.03059

**Published:** 2020-01-28

**Authors:** Miriam M. Fonseca, Marcela Davoli-Ferreira, Flávia Santa-Cecília, Rafaela M. Guimarães, Francisco F. B. Oliveira, Ricardo Kusuda, David W. Ferreira, José C. Alves-Filho, Fernando Q. Cunha, Thiago M. Cunha

**Affiliations:** ^1^Department of Pharmacology, Center for Research in Inflammatory Diseases (CRID), Ribeirão Preto Medical School, University of São Paulo (USP), Ribeirão Preto, Brazil; ^2^Graduate Program in Basic and Applied Immunology, Ribeirão Preto Medical School, University of São Paulo (USP), Ribeirão Preto, Brazil

**Keywords:** neuropathic pain, cytokines, IL-27, IL-10, glial cells, macrophages

## Abstract

Neuroimmune–glia interactions have been implicated in the development of neuropathic pain. Interleukin-27 (IL-27) is a cytokine that presents regulatory activity in inflammatory conditions of the central nervous system. Thus, we hypothesized that IL-27 would participate in the neuropathic pain process. Here, we found that neuropathic pain caused by peripheral nerve injury (spared nerve injury model; SNI), was enhanced in IL-27-deficient^(−/−)^ mice, whereas nociceptive pain is similar to that of wild-type mice. SNI induced an increase in the expression of IL-27 and its receptor subunit (*Wsx1*) in the sensory ganglia and spinal cord. IL-27 receptor was expressed mainly in resident macrophage, microglia, and astrocytes of the sensory ganglia and spinal cord, respectively. Finally, we identify that the antinociceptive effect of IL-27 was not observed in IL-10^−/−^ mice. These results provided evidence that IL-27 is a cytokine produced after peripheral nerve injury that counteracts neuropathic pain development through induction of the antinociceptive cytokine IL-10. In summary, our study unraveled the role of IL-27 as a regulatory cytokine that counteracts the development of neuropathic pain after peripheral nerve damage. In conclusion, they indicate that immunotherapies based on IL-27 could emerge as possible therapeutic approaches for the prevention of neuropathic pain development after peripheral nerve injury.

## Introduction

Chronic neuropathic pain is caused by diseases or lesions affecting the somatosensory nervous system. Often resistant to different types of therapies, neuropathic pain affects ~3–17% of the chronic pain population in the world (1). Many studies have shown that neuroimmune response in nociceptive pathway has an important role for the development of neuropathic pain (2–4). Different forms of injury to the peripheral nervous systems, evoked by traumatic, metabolic, or toxic events, can lead to abnormal neuronal response from the site of damage to the sensory ganglia and then the central nervous system (CNS), inducing an intense neuroinflammation response in these sites (5, 6). Non-neuronal cells, including satellite glial cells, macrophages, microglia, and astrocytes, along the nociceptive pathway can be activated, which influence the neuronal excitability and consequently participate in the establishment of chronic pain states (7–9). In the local of nerve injury, sensory ganglia, and spinal cord, non-neuronal cells can release a diverse range of proinflammatory mediators, which may directly or indirectly amplify pain signaling (10, 11). On the other hand, in order to control the inflammatory and nociceptive responses, these cells can also release anti-inflammatory mediators, such as IL-4, IL-10, IL-13, and transforming growth factor beta (TGF-β) and pro-resolving mediators such as resolvins, lipoxins, protectins, and maresins, which regulate the response to nerve damage and might attenuate pain behavior in animal models of chronic pain (12–16). Another cytokine that appears to control the inflammatory response in the periphery and CNS is interleukin-27 (IL-27) (17).

IL-27 is a member of the IL-6/IL-12 heterodimeric cytokine family, formed by two subunits, Epstein–Barr virus-induced gene 3 (EBI3) and p28. It binds to a receptor composed of gp130 (shared with other cytokine receptors, including IL-6R) and IL-27Rα (also known as WSX-1 or TCCR), which is specific for IL-27 (18, 19). IL-27 is generally produced by antigen-presenting cells, such as macrophages and dendritic cells; however, some studies suggest that it can be released by astrocytes and microglia in CNS (20, 21), and the signaling for IL-27 subunit production is induced by immune stimuli and different pathogens (18, 22). IL-27 is remarkably characterized by immunosuppressive and anti-inflammatory functions mediated by the production of IL-10 and suppression of IL-17 expression (20, 23, 24). IL-27 can suppress the differentiation of naïve T cells toward IL-17-producing T cells (Th17). It also promotes differentiation of activated T cells to T cells able to secrete IL-10 in an antigen-specific manner, named type 1 regulatory (Tr1) T cells (25, 26).

The immunomodulatory activity of IL-27 has been demonstrated in several *in vivo* models. WSX-1-deficient mice develop an excessive inflammatory response during infections and in autoimmune disease models (27, 28). In a model of infection in the CNS induced by the JHM strain of mouse hepatitis virus, IL-27 promotes the production of IL-10, which is essential for controlling inflammation response (29). In addition, in a murine model of experimental encephalomyelitis (EAE), treatment with recombinant IL-27 delays the onset of EAE and improves the clinical signs of the disease (30). Furthermore, IL-27 has demonstrated potential therapeutic action in the rheumatoid arthritis model (31–33). Although many studies are showing the importance of IL-27 in neuroimmune-mediated diseases, there is no study investigating its role in the pathophysiology of neuropathic pain. Herein, we showed that IL-27 is upregulated in the dorsal root ganglia (DRGs) and spinal cord of mice after peripheral nerve injury (spared nerve injury, SNI). Moreover, we showed that IL-27 counteracted the development of neuropathic pain through the induction of IL-10 production.

## Methods

### Animals

The experiments were performed in C57BL/6 wild-type (WT) male mice (6–8 weeks old) and C57BL/6 mice deficient (^−/−^) in the following proteins: IL-27 (EBI3) (34) and IL-10 (35), as well as in transgenic animals expressing the green fluorescent protein (GFP) in cells that express CX3C chemokine receptor 1 (CX3CR1^GFP/+^) (36). Local colonies of transgenic mice were then established and maintained on a C57BL/6 background at the animal care facility of Ribeirão Preto Medical School, University of São Paulo. The controls and transgenic mice were not littermates. The animals were taken to the testing room at least 1 h before the experiments. Food and water were available *ad libitum*. All behavioral tests were performed between 8:00 a.m. and 5:00 p.m.

### Drugs and Chemicals

The following drugs were obtained from the sources indicated: recombinant mouse IL-27 (NS0-expressed) protein (IL-27r) (2799-ML-010, R&D, Minneapolis, USA) and diazepam (D0899, Sigma, St Louis, MO, USA). Acetone (Fisher Chemical, Gell, Belgium). Formalin solution (1%) was prepared using 37% formaldehyde (Fisher Chemical Gell, Belgium) in 0.9% sodium chloride saline solution (Baxter hospitalar, São Paulo, Brazil). To prepare 1 L of phosphate-buffered saline (PBS), 0.1 M was used with 9 g of NaCl, 0.122 g of KH_2_PO_4_, and 0.814 g of Na_2_HPO_4_ (Sigma-Aldrich, St. Louis, NO, USA) in distilled water (pH = 7.4).

### Neuropathic Pain Model

A model of persistent peripheral neuropathic pain, the spared nerve injury (SNI), was induced as previously described (37). Briefly, under isoflurane anesthesia, the sciatic nerve and their three terminal branches (the sural, common peroneal, and tibial nerves) were exposed at lower-thigh level by blunt dissection through the biceps femoris muscle. The common peroneal and tibial nerves were tightly ligated with 6.0 silk, while the sural nerve was spared. The nerves distal to the ligature were sectioned. The experimental control group consisted of false-operated animals (Sham). In these animals, there was exposure of the sciatic nerve similarly to the SNI group, but without any manipulation of nerves. Muscle and skin were closed in layers. Then, mechanical pain hypersensitivity and cold allodynia were evaluated on specific days.

### Behavioral Nociceptive Tests

An investigator blinded to group allocation performed all the behavior tests.

### Formalin Assay

We assessed formalin-evoked nociception by injection of 20 μl of formalin (1%) into the dorsal surface of the right hind paw of two groups of male mice: IL-27^−/−^ and WT-naive mice. The time in seconds spent licking or flinching the injected paw was recorded and expressed as the total time of nociceptive behaviors in the early phase (0–10 min) and late phase (10–50 min) after formalin injection (38).

### Hot Plate Test

The noxious thermal thresholds of the hind paws were examined in IL-27^−/−^ and WT-naive mice via hot plate test using an electrically heated surface (Ugo Basile, model 35100, Gemonio VA, Italy) at different temperatures (48, 50, 52, and 56°C). For each specified temperature, each animal was placed on the heated plate, and time responses to the thermal stimulus (jumping, withdrawal, and licking of the hind or front paws) were recorded in seconds. The cutoff time used was 20 s to avoid possible injury in the animal paws (39).

### Acetone Test

In different days after SNI, IL-27^−/−^, and WT mice were placed in a clear plastic box with a wire mesh floor and allowed to habituate for 30 min prior to testing. Then, 50 μl fluid (acetone) was sprayed on the plantar surface of the right hind paw using a syringe of 1 ml (Tuberculin slip tip, BD, Franklin Lake, NJ, USA). Paw withdrawal response, defined as flinching, licking, or biting of the limb, was measured within 1 min after the application of acetone (40). The acetone test was measured after the paw mechanical withdrawal threshold test was done.

### Mechanical Nociception Test

The mechanical nociceptive threshold was evaluated in WT, IL-27^−/−^, and IL-10^−/−^ mice that were placed on an elevated wire grid, and the plantar surface of the ipsilateral hind paw was stimulated perpendicularly with a series of von Frey filaments (Stoelting, Chicago, IL, USA), with logarithmically increasing stiffness (0.008–2.0 g) (41). The withdrawal frequency was calculated as a percentage of withdrawals after 10 applications of 0.008 g von Frey filament in the right hind paw.

### Rota-rod Test

To discard possible non-specific muscle relaxant or sedative effects of recombinant mouse IL-27, mice motor performance was evaluated on the rota-rod test (42). The apparatus consisted of a bar with a diameter of 2.5 cm, subdivided into six compartments by disks 25 cm in diameter (Ugo Basile, Model 7600). The bar rotated at a constant speed of 22 rotations per minute. WT-naive animals were selected 24 h previously by eliminating those mice that did not remain on the bar for two consecutive periods of 120 s. Then, those animals were treated intrathecally with vehicle (saline) or recombinant IL-27 (100 ng) in a final volume of 5 μl using a BD Ultra-Fine® (29G) insulin syringe (BD, Franklin Lakes, NJ, USA). Diazepam (5 mg/kg, intraperitoneal) was used as a positive control. To inject diazepam solution, 1 ml sub-Q syringe and needle 26G x 5/8 (BD, Franklin Lakes, NJ, USA) were used. The cutoff time used was 120 s.

### Intrathecal Injection

Under isoflurane (2%) anesthesia, recombinant IL-27 (100 ng) or vehicle (saline) was administered intrathecally (i.t.) in WT and IL-10^−/−^ mice. The technique used for intrathecal injection was described by Papir-Kricheli et al. (43), modified by holding the mice securely in one hand by the pelvic girdle and inserting a BD Ultra-Fine® (29G) insulin syringe (BD, Franklin Lakes, NJ, USA) directly on the subarachnoid space (close to L4–L5 segments) of the spinal cord. A sudden lateral movement of the tail indicated proper placement of the needle in the intrathecal space. For all administrations, 5-μl volume was used. Then, the syringe was held in the specific position for a few seconds and progressively removed to avoid any outflow of the drug.

### Quantitative Real-Time PCR

At the indicated times after nerve injury, mice were anesthetized and then perfused with 0.1 M PBS. DRGs and spinal cord, ipsilateral to the lesion, were collected from the region correspondent to lumbar segments (L3–L5) and homogenized in TRI Reagent® (Life Technologies Corporation, Carlsbad, CA, USA) reagent at 4°C. Then, total cellular mRNA was purified from whole tissues, according to the manufacturer's instructions. After FACS sorting procedure, ~25,000 cells were used to isolate mRNA using RNeasy micro kit® (Qiagen, Hiden, Germany). To convert the mRNA in cDNA, high-capacity cDNA Reverse Transcription Kit (Thermo Fisher Scientific, Graiciuno, Vilnius, LT) was used. The level of each gene was normalized to the levels of the mouse *Gapdh* gene, and the results were analyzed by the method of quantitative relative expression 2^−ΔΔ*CT*^ as previously described (44). Primer pairs for mouse *Gapdh, Aif1, Gfap, Tnf*, *Il1b, Ebi3, p28, Wsx1, Il12p35*, and *Il10* were as follows:

*Gapdh* fwd: 5′-CATCTTCTTGTGCAGTGCCA-3′
*Gapdh* rev: 5′-CGGCCAAATCCGTTCAC-3′
*Aif1* fwd: 5′-TGAGGAGCCATGAGCCAAAG-3′
*Aif1* rev: 5′-GCTTCAAGTTTGGACGGCAG-3′
*Gfap* fwd: 5′-AGGGCGAAGAAAACCGCATCACC-3′
*Gfap* rev: 5′-TCTAAGGGAGAGCTGGCAGGGCT-3′
*Tnf* fwd: 5′-TGTGCTCAGAGCTTTCAACAA-3′
*Tnf* rev: 5′-CTTGATGGTGGTGCATGAGA-3′
*Il1b* fwd: 5′-TGACAGTGATGATGAGAATGACCTGTTC-3′
*Il1b* rev: 5′-TTGGAAGCAGCCCTTCATCT-3′
*Il27* (*Ebi3*) fwd: 5′-TGCCATGCTTCTCGGTATCC-3′
*Il27* (*Ebi3*) rev: 5′-AGGGTCCGGCTTGATGATTC-3′
*Il27* (*p28*) fwd: 5′-GGCTATGTCCACAGCTTTGCT-3′
*Il27* (*p28)* rev: 5′-CGAAGTGTGGTAGCGAGGAA-3′
*Wsx1*: Taqman®: Mm00497259_m1
*Il12p35* fwd: 5′-AAGACATCACACGGGACCAAA-3′
*Il12p35* rev: 5′-CAGGCAACTCTCGTTCTTGTGTA-3′
*Il10* fwd: 5′-AACAAAGGACCAGCTGGACAAC-3′
*Il10* rev: 5′-GCAACCCAAGTAACCCTTAAAGTC-3′

### Immunofluorescence

At day 10 after surgery, WT and CX3CR1^GFP/+^ mice were deeply anesthetized with ketamine and xylazine and perfused transcardially with phosphate buffer 0.1 M, followed by fresh 4% paraformaldehyde (PFA) in PBS 0.1 M (pH 7.4). After the perfusion, segments of spinal cord lumbar correspondent L3, L4, and L5 were dissected out, post-fixed for 2 h in PFA, and then replaced with 30% sucrose overnight. Transverse spinal sections (free-floating, 60 μm) were cut in a cryostat. The floating sections were used for immunofluorescence assays as previously described (45). Then, the sections were incubated overnight at 4°C with polyclonal primary antibodies: anti-WSX-1 (1:250) (5996—Abcam), anti-GFAP (1:500) conjugated with alexa fluor 488 (MAB 3042X—Millipore), anti-NeuN (1:250) (MAB377—Millipore), and anti-GFP (1:500) conjugated with FITC (ab6662—Abcam), for the tissue from CX3CR1^GFP/+^ mice. After washing, the sections were then incubated with the appropriate secondary antibody solution for 2 h at room temperature; all secondary solutions were diluted 1:500: Alexa fluor 594, Alexa fluor 488, or Alexa fluor 647 (Invitrogen). The sections were washed with PBS as described earlier, mounted on glass slides, and covered with coverslips with Fluromount^TM^ Aqueous Mounting Medium (Sigma). The sections of spinal cord were acquired using a SP5 confocal laser scanning microscope (Leica, Wetzlar, Germany). Colocalization was ensured with confocal Z stacks at 1-μm intervals and visualization in three-dimensional orthogonal planes.

### Cell Sorting

Cell sorting of CD11b^+^ and CD45^−^ cells from DGRs was performed as described previously (46). Briefly, the ipsilateral DRGs (L3, L4, and L5 pooled from eight animals) were collected at day 10 from WT or IL-27^−/−^ mice submitted to SNI or Sham surgery, as well as from WT mice submitted to SNI and treated for five consecutive days (starting on day 10 up to day 14) with recombinant IL-27 (100 ng/in a final volume of 5 μl) or vehicle (i.t). DRGs were incubated in 5 ml of RPMI medium containing 2 mg/ml of collagenase type II (Sigma Aldrich) for 1 h at 37°C. After this time, the tissues were passed through a cell strainer (40 μm), followed by centrifugation with RPMI medium containing 10% fetal bovine serum (FBS, Gibco). Cells obtained were resuspended in 0.1 M PBS containing 10% of rabbit serum, to avoid non-specific background staining, and specific monoclonal antibodies for 30 min at 4°C. The following monoclonal antibodies (BD Biosciences) were used for staining: CD45-BV421 (clone 30-F11), Ly6G-FITC (clone 1A8), and CD11b-APCCy7 (clone M1/70); the antibodies were diluted 1:250. Cells were isolated using FACSAria III (BD Biosciences), and then the pellets obtained were resuspended in lysis buffer to isolate mRNA. For all of the experiments, sample purity was ~70% for CD11b+ Ly6G- cells and ~90% for CD45- cells. The data were analyzed using FlowJo 10 software (Treestar, Ashland, USA). The gating strategy used to perform cell sorting from sensory ganglia is depicted in Figure S1.

### Cytokine Measurement by ELISA

At 7 and 10 days after nerve injury, mice were anesthetized and then perfused with 0.1 M PBS. The ipsilateral DRGs and lumbar segments of the spinal cord (L3, L4, and L5 pooled from two animals) from WT or IL-27^−/−^ mice were collected and homogenized in lysis buffer containing protease inhibitors (to each 10 mg of tissue, 100 μl of lysis buffer was used); the supernatant without dilution was used to measure IL-10. Mouse DuoSet ELISA kit to IL-10 was purchased from R&D Systems and performed as instructed by the manufacturer. The results are expressed as picogram (pg) of cytokines per milligram (mg) of tissue protein. The protein concentration of the lysate was determined using a Pierce^TM^ bicinchoninic acid assay protein assay kit (Thermo Scientific, Rockford, IL, USA).

### Data Analyses and Statistics

Data are reported as means ± SEM. The normality of the distribution of data was analyzed by D'Agostino and Pearson tests. Two-way ANOVA followed by Bonferroni's *t*-test was used to compare the groups and doses at different times (curves) when the responses (nociception) were measured. The factors analyzed were treatment, time, and time vs. treatment interaction. Alternatively, if the responses (nociception, protein expression, mRNA expression) were measured only once after stimulus injection, the differences between responses were evaluated by one-way ANOVA followed by Bonferroni's *t*-test (for three or more groups), comparing all pairs of columns. For comparisons of groups across two groups, an unpaired Student *t*-test was used. *P* < 0.05 were considered significant. Statistical analysis was performed with GraphPad Prism (GraphPad Software, San Diego, CA, USA).

## Results

### IL-27 Counteracts the Development of Neuropathic Pain but Is Not Involved in Basal Control of Nociception Threshold

**Figure 1:**
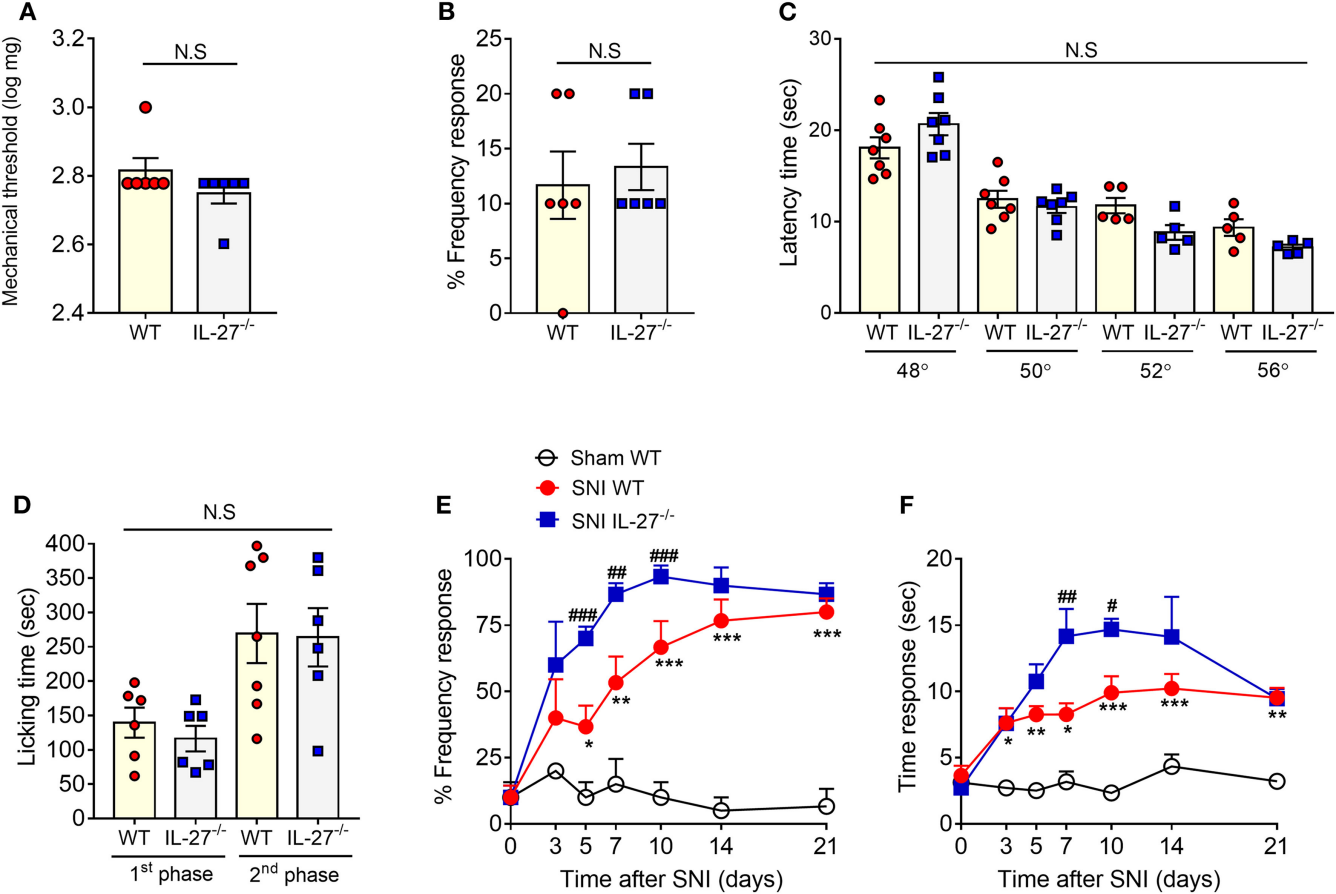
IL-27 signaling is not involved in nociceptive pain but protects the animals in neuropathic pain condition. Different behavior tests were realized in WT and IL-27^−/−^ mice: mechanical nociceptive threshold using von Frey filaments **(A)** (*n* = 6). Percentage of withdrawal frequency using von Frey filaments **(B)** (*n* = 6). Thermal nociceptive threshold using hot plate test at different temperatures **(C)** (*n* = 6). Total duration (seconds) of nociceptive behavior for 0–10 min (first phase) and for 10 up to 50 min (second phase) after administration of formalin in the paw **(D)** (*n* = 6). Mechanical nociception test using percentage of withdrawal frequency **(E)** (*n* = 8) and acetone test **(F)** (*n* = 8), in which values represent withdrawal threshold before (day 0) and up to 21 days after nerve injury (SNI) or in sham-operated mice. Data are presented as means ± S.E.M. *P < 0.05, **P < 0.01, ***P < 0.001 vs. Sham-WT and ^#^*P* < 0.05, ^##^*P* < 0.01, ^###^*P* < 0.001 vs. SNI-WT. Data were analyzed by *t*-test **(A–D)** and two-way ANOVA followed by Bonferroni post-test **(E,F)**.

We first characterized the physiological nociceptive response of IL-27^−/−^ mice. WT and IL-27^−/−^ mice were assessed behaviorally after acute peripheral application of different stimuli. IL-27^−/−^ mice exhibited the same intensity of mechanical pain threshold (using von Frey filament) and thermal pain threshold (temperatures of 48, 50, 52, and 56°C) compared to WT mice (Figures 1A–C). Furthermore, IL-27^−/−^ mice also showed no difference in nociceptive behaviors in the early and late phase of the formalin test compared to WT mice (Figure 1D). These data indicate that, under normal physiological conditions, IL-27 is not involved in acute nociception. Next, we determined whether IL-27 plays a role in the development of chronic neuropathic pain induced by SNI. The injured WT mice (WT-SNI) showed an increase in mechanical hypersensitivity (more percentage of frequency response), starting on day 3 and reaching the maximal response at day 10, which persists until day 21 compared to sham-operated WT mice (WT-Sham). In contrast, IL-27^−/−^ mice submitted to SNI (IL-27^−/−^-SNI) displayed a significant increase in mechanical hypersensitivity after nerve injury on day 5 until day 10 compared to WT-SNI mice (Figure 1E). Similarly, WT-SNI mice showed an increase in cold hypersensitivity, starting on day 3 until day 21, compared to Sham-WT mice. Furthermore, IL-27^−/−^-SNI animals also showed increased levels of cold hypersensitivity when compared to WT-SNI mice (Figure 1F). Taken together, these data indicate that IL-27 signaling might be negatively controlling the development of neuropathic pain but is not involved in the detection of mechanical, thermal, and chemical nociception in basal conditions.

### Peripheral Nerve Injury Triggers an Increase in the Expression of IL-27 and Its Receptor-Specific Subunit, WSX-1, in Spinal Cord and Dorsal Root Ganglia

**Figure 2:**
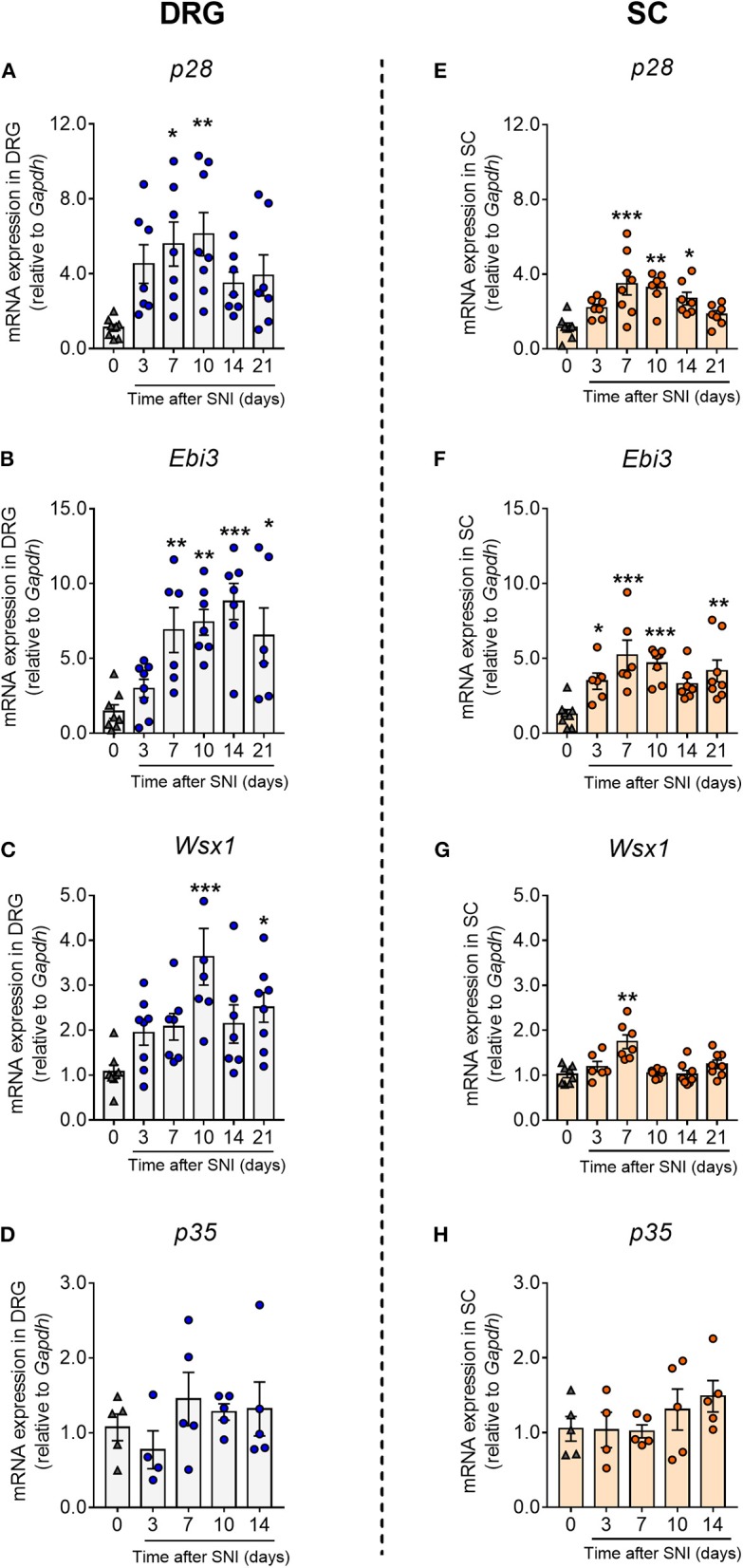
Peripheral nerve injury upregulated the expression of IL-27 and its receptor in spinal cord and dorsal root ganglia. Real-time PCR analysis on days 3, 7, 10, 14, and 21 after SNI or sham-operated (0) in WT mice: *p28* **(A)**, *Ebi3* **(B)***, Wsx1* **(C)**, and *Il12p35* **(D)** in DRGs; *p28* **(E)**, *Ebi3* **(F)**, *Wsx-1* **(G)**, and *Il12p35* **(H)** in spinal cord, relative expression to *Gapdh* levels (*n* = 5–8). Data are presented as means ± S.E.M. *P < 0.05, **P < 0.01, ***P < 0.001 vs. Sham-operated. Data were analyzed by one-way ANOVA followed by Bonferroni post-test.

To explore the role of IL-27 signaling in the development of neuropathic pain, we evaluated the levels of mRNA expression of IL-27 subunits (*p28* and *Ebi3*) and *Wsx1*, a specific subunit of its receptor, in ipsilateral lumbar DRGs (L3–L5) and spinal cord of WT animals after SNI induction. We detected that *p28* expression was upregulated between days 7 and 14 in the DRGs and spinal cord of WT-SNI mice compared to WT-Sham mice (Figures 2A,E). *Ebi3* expression increased either in DRGs and spinal cord after SNI at all times evaluated (Figures 2B,F). *Wsx1* expression was also upregulated in the DRGs (10 days after SNI) and at day 7 in the spinal cord of WT-SNI mice compared to WT-Sham mice (Figures 2C,G). To exclude the participation of IL-35 cytokine because it is formed by EBI3 and IL12p35, we analyzed the gene expression of *Il12p35* in the same tissues; however, we did not detect any difference in all times analyzed (Figures 2D,H). Altogether these results indicate that after peripheral nerve injury, IL-27 and its specific receptor subunit, but not IL-35, are upregulated in the sensory ganglia and spinal cord.

### WSX-1 Is Expressed in Microglia and Astrocytes in the Spinal Cord and in the Macrophages of DRGs After Peripheral Nerve Injury

**Figure 3:**
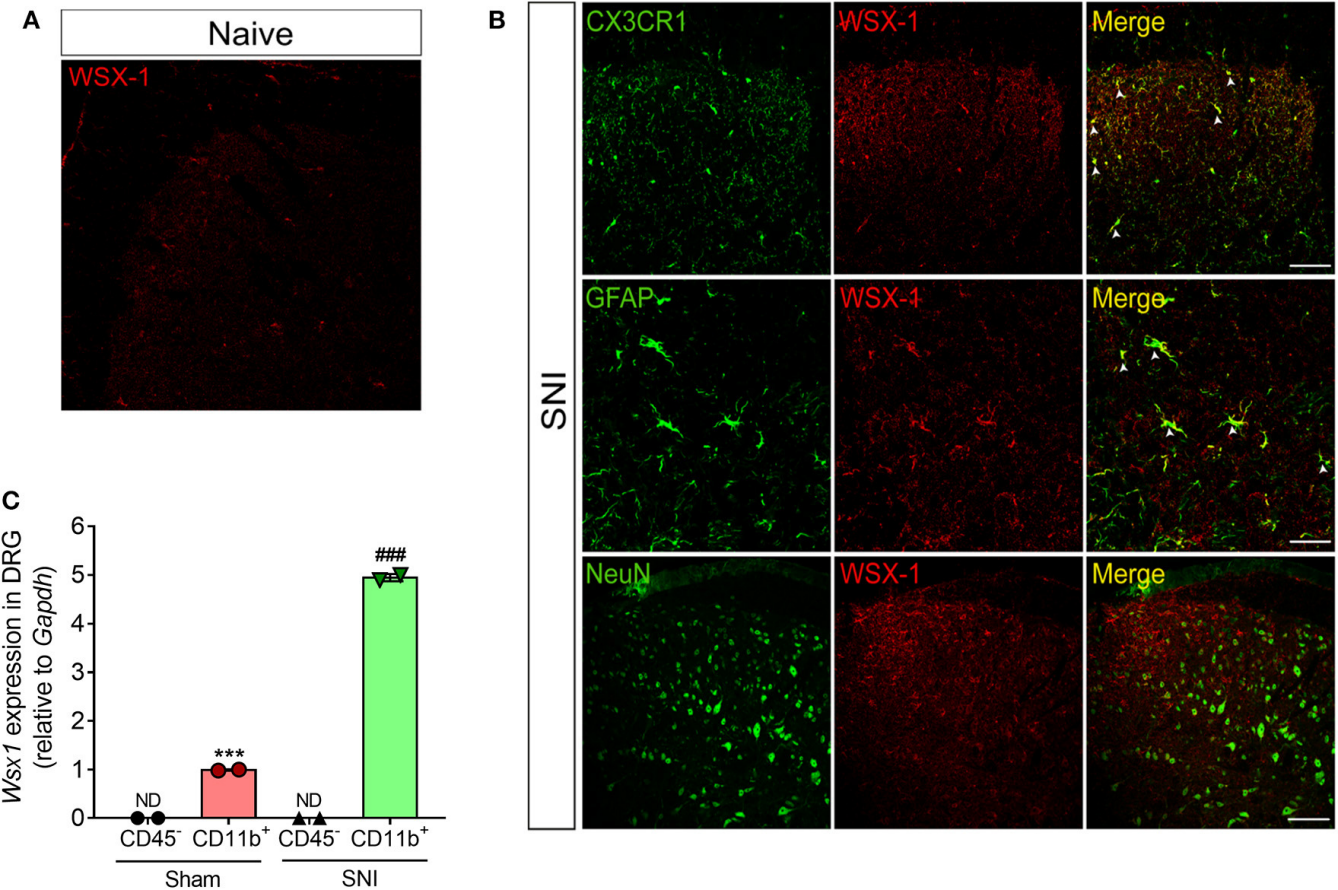
Peripheral nerve injury induces WSX-1 expression in glial cells in spinal cord and in macrophage of DRGs. Representative confocal images of spinal cord at day 10 after SNI in CX3CR1^GFP/+^ and WT mice. **(A)** Spinal cord from CX3CR1^GFP/+^-naive mice labeled with WSX-1 (*red*). **(B)** Sections from CX3CR1^GFP/+^ and WT mice were double-labeled marked with WSX-1 (*magenta* or *red*; middle panels), GFAP (*green*; left and middle panels), or NeuN (*green*; left panel, above). The merged images are shown in the right panels (*yellow* indicates double-positive cells, followed by *arrows*). Scale bars, 25 uM (*n* = 4). **(C)** Real-time PCR analysis of *Wsx1* expression relative to *Gapdh* levels in CD45^−^ and CD11b^+^ cells isolated, by FACS sorting, from DRGs of SNI or Sham-operated WT mice at day 10 after surgery (*n* = 2, pool from eight animals for each). Data are presented as means ± S.E.M. ***P < 0.001 vs. CD45^−^ from Sham. ^###^*P* < 0.001 vs. CD11b^+^ from Sham. ND, not detected. Data were analyzed by one-way ANOVA followed by Tukey post-test.

Next, we sought to analyze which cell type might be expressing the IL-27 receptor in the sensory ganglia and spinal cord after peripheral nerve injury. In naïve animals, the immunoreactivity for WXS-1 is very low in the dorsal horn of the spinal cord (Figure 3A), corroborating the mRNA data; however, WSX-1 expression is upregulated in the spinal cord at 10 days after SNI. WSX-1 is expressed in the spinal cord cells that also express CX3CR1 and GFAP, but not NeuN (Figure 3B). Thus, these data suggest that after peripheral nerve injury, WXS-1 expression might be upregulated in microglia and astrocytes, but not in neurons of the spinal cord.

In an attempt to evaluate in which cell type WSX-1 might be expressed in sensory ganglia, at day 10 after SNI or Sham surgeries, DRGs were harvested and CD45^−^ and CD11b^+^ Ly6G^−^ cells (macrophages) were purified using FACS sorting. Then, we analyzed *Wsx1* gene expression in these cells. It was found that *Wsx1* mRNA was only detected in CD11b^+^ Ly6G^−^ cells, and the expression was upregulated after SNI in these cells compared to cells from Sham-operated mice (Figure 3C). These results suggest that, in the sensory ganglia, *Wsx1* is mainly expressed in macrophages, and peripheral nerve injury induces an increase in its expression.

### The Impact of IL-27 Deficiency in the Activation of Immune/Glial Cells and Cytokine Production in the DRGs and Spinal Cord After Peripheral Nerve Injury

**Figure 4:**
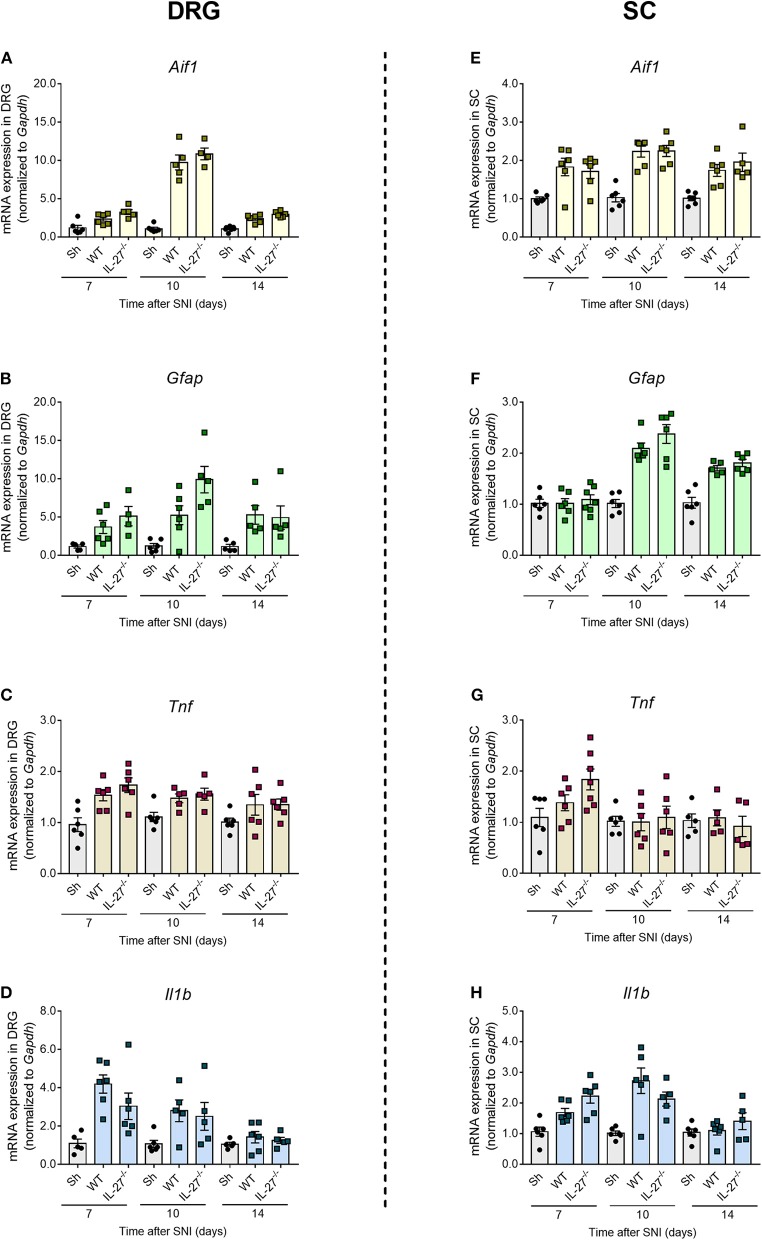
Activation of immune/glial cells and cytokine expression in the DRGs and spinal cord after peripheral nerve injury in the absence of IL-27. Real-time PCR analysis of the expression of glial markers and pro-inflammatory cytokines relative to *Gapdh* at days 7, 10, and 14 after SNI or Sham surgery in WT and IL-27^−/−^ mice: **(A)** *Aif1*, **(B)** *Gfap*, **(C)** *Tnf*, and **(D)** *Il1b* expressions in DRGs, **(E)** *Aif1*, **(F)** *Gfap*, **(G)** *Tnf*, and **(H)** *Il1b* expressions in spinal cord (*n* = 7). Data are presented as means ± S.E.M. Data were analyzed by one-way ANOVA followed by Bonferroni post-test.

Peripheral nerve injury induces the activation of resident macrophages in the sensory ganglia and glial cells (microglia and astrocytes) in spinal cord that account for the development of neuropathic pain through the production of pro-inflammatory cytokines such as TNF and IL-1β (2, 4, 47, 48). Since the expression of IL-27 receptor is mainly observed in glial/immune cells of the spinal cord and DRGs after peripheral nerve injury, we hypothesize that the role of IL-27 in counteracting neuropathic pain might be through regulating these neuroimmune–glia processes. Thus, we analyzed the gene expression of *Aif1* (IBA-1) and *Gfap* as indicators of the macrophages/microglial cell and satellite glial/astrocyte activation in the DRGs and spinal cord, respectively. In all times and tissues evaluated, the expression of *Aif1* and *Gfap* in IL-27^−/−^-SNI mice was not different from WT-SNI mice (Figures 4A,E,B,F). Furthermore, the expression of *Tnf* and *Il1b* was also not different between IL-27^−/−^-SNI and WT-SNI mice (Figures 4C,D,G,H).

**Figure 5:**
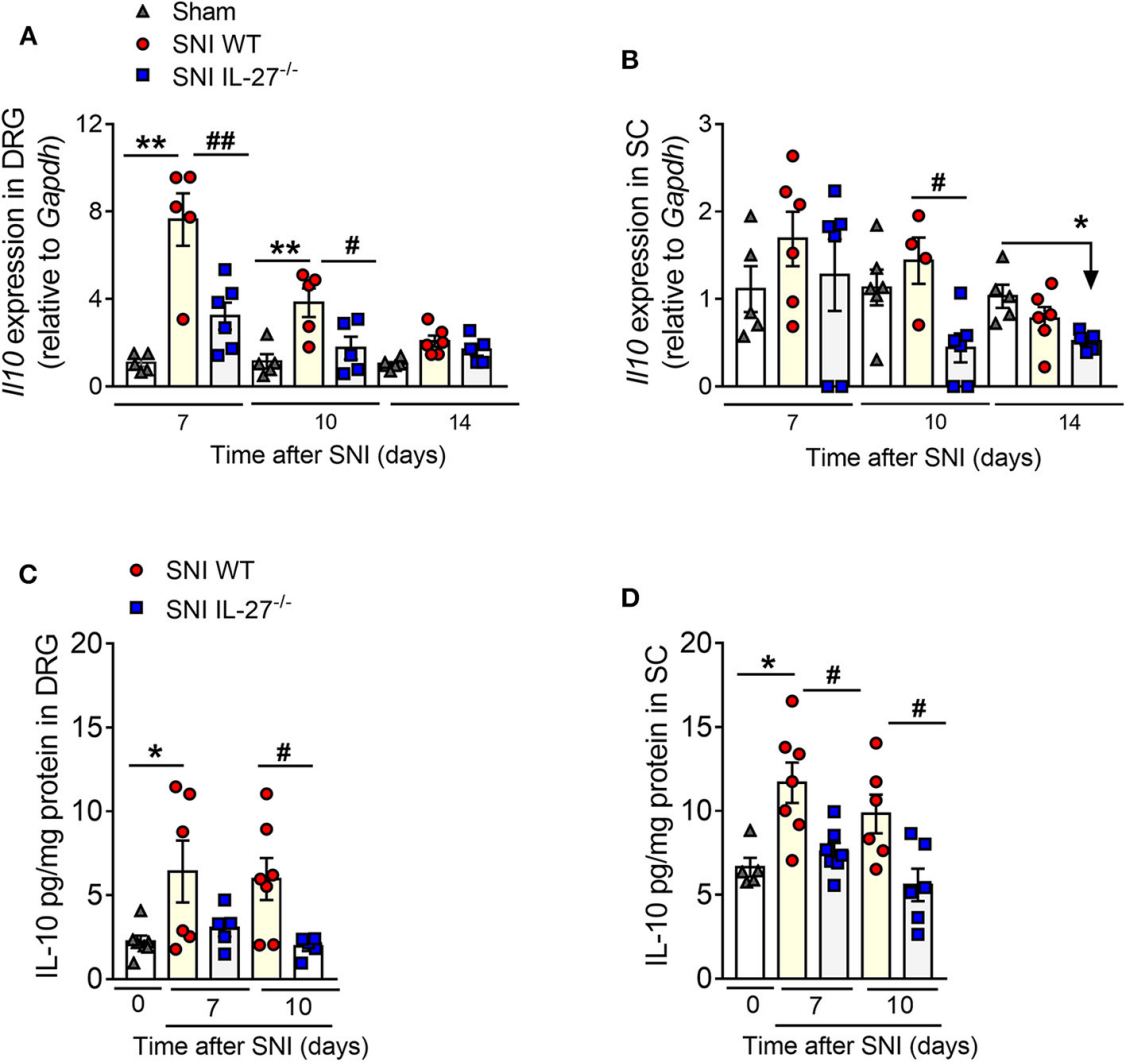
The impact of IL-27 deficiency in anti-inflammatory cytokine expression after peripheral nerve injury. WT and IL-27^−/−^ mice were submitted to SNI or Sham surgeries, and at days 7, 10, and 14, tissues were collected to real-time PCR analysis: **(A,B)** *Il10* expression relative to *Gapdh* in DRGs and in the spinal cord, respectively. **(C,D)** Level of IL-10 measured by ELISA in DRGs and in the spinal cord, respectively, on days 10 and 14 after SNI or Sham-operated (day 0) in WT and IL-27^−/−^ mice. Data are presented as means ± S.E.M (*n* = 5–7). *P < 0.05 and **P < 0.01 vs. Sham-WT. ^#^*P* < 0.05 and ^##^*P* < 0.01 vs. SNI-WT **(A,B)**. *P < 0.05 and **P < 0.01 vs. Sham-operated (day 0). ^#^*P* < 0.05 and ^##^*P* < 0.01 vs. SNI-WT **(C,D)**. Data were analyzed by one-way ANOVA followed by Bonferroni post-test.

There is evidence that the immunoregulatory role of IL-27 is dependent on its ability to induce IL-10 production (23, 28, 29). Furthermore, IL-10 is a well-known anti-nociceptive cytokine that reduces the development of pathological pain, including neuropathic pain (49–51). Based on these evidences, we analyzed next the interference of IL-27 deficiency on IL-10 production in DRGs and spinal cord after peripheral nerve injury. Notably, it was found that the expression of IL-10 (mRNA and protein) in the DRGs and spinal cord was reduced in IL-27^−/−^-SNI mice when compared to WT-SNI mice (Figures 5A–D). Collectively, these results indicate that IL-27 is a triggering mechanism of IL-10 production after peripheral nerve injury; however, it has minimum effect on immune/glial cell activation and pro-nociceptive cytokine production.

### Exogenous IL-27 Reduced Neuropathic Pain Through the Induction of IL-10

**Figure 6:**
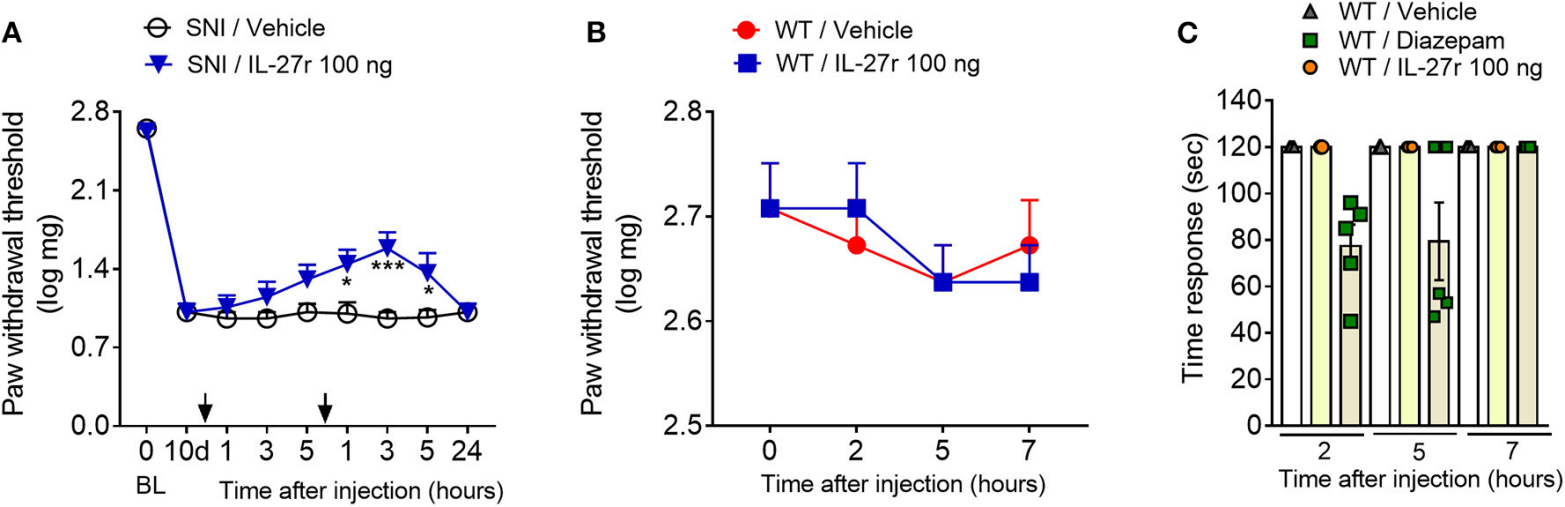
Recombinant IL-27 intrathecally injected partially reverses mechanical hypersensitivity after peripheral nerve injury. **(A)** WT animals were submitted to SNI surgery and at day 10 were treated i.t. with recombinant IL-27 (IL-27r) or vehicle, and the treatments were repeated after ~5 h; mechanical threshold was evaluated using von Frey filaments (*n* = 8). **(B)** Effect of recombinant IL-27 in the basal mechanical threshold in WT-naive animals (*n* = 6). **(C)** Rota-rod test was realized to discard possible non-specific muscle relaxant or sedative effects of recombinant IL-27 in WT-naive animals (*n* = 5). Data are presented as means ± S.E.M. **(A)** *P < 0.05, ***P < 0.001 vs. SNI-vehicle. **(C)** *P < 0.05 vs. WT-vehicle. Data were analyzed by two-way ANOVA followed by Bonferroni post-test.

Since IL-27^−/−^ animals presented higher signs of neuropathic pain, it might indicate that exogenous IL-27 would be a useful tool to reduce neuropathic pain. In this context, we analyzed whether the intrathecal administration of recombinant IL-27 could affect the mechanical pain hypersensitivity induced by SNI. Previously, we conducted a long series of experiments in an attempt to find the best dose and time for recombinant IL-27 treatments (data not shown). We found that on day 10 after SNI induction, the treatment of animals intrathecally with recombinant IL-27 (100 ng/site), twice a day, was able to reduce mechanical pain hypersensitivity (Figure 6A). It is noteworthy that this dose of recombinant IL-27 neither changed the mechanical withdrawal threshold of naive mice nor caused motor impairment, discarding possible non-specific muscle relaxant or sedative effects of recombinant IL-27 (Figures 6B,C).

**Figure 7:**
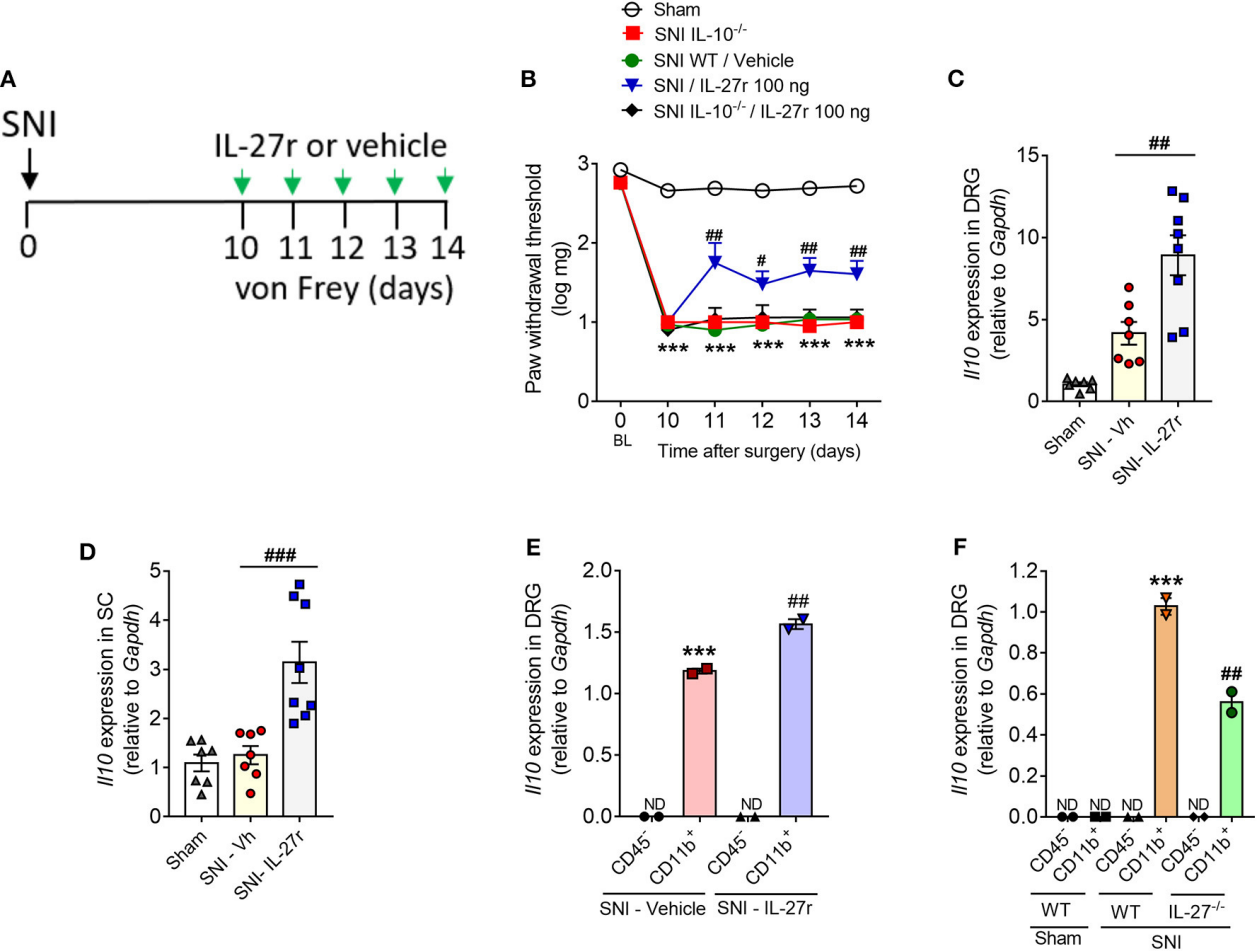
Exogenous IL-27 reduced neuropathic pain through the induction of IL-10. **(A)** WT and IL-10^−/−^ mice were submitted to SNI (day 0), and on day 10 up to day 14, different groups of animals were treated with recombinant IL-27 or vehicle (i.t) daily. **(B)** Paw withdrawal thresholds were evaluated using von Frey filament before (day 0) and up to 14 days after SNI (*n* = 8). ***p < 0.001 vs. Sham-operated mice; ^#^*p* < 0.05 and ^##^*p* < 0.01 vs. WT-SNI. Real-time PCR analysis of *Il10* expression relative to *Gapdh* in WT mice submitted to SNI or Sham surgeries and treated with recombinant IL-27 (SNI-IL27r) or vehicle (SNI-Vh) on day 10 until day 14 **(C)** in DRGs and **(D)** in the spinal cord (*n* = 7–8). ***P < 0.001 vs. Sham-operated; ^##^*P* < 0.05 and ^###^*P* < 0.001 vs. SNI-Vh. **(E)** Using the same protocol of treatment, CD45^−^ and CD11b^+^ cells were isolated from DRGs, by FACS sorting, and levels of *Il10* relative to *Gapdh* expression were quantified using real-time PCR (*n* = 2, pool from eight animals for each). ***P < 0.001 vs. CD45^−^ SNI-vehicle. ^##^*P* < 0.05 vs. CD11b^+^ from SNI-vehicle; *ND* not detected. **(F)** At 10 days after SNI or Sham surgeries, cells were isolated from DRGs of WT and IL-27^−/−^ and real-time PCR analysis of *Il10* expression relative to *Gapdh* was realized (*n* = 2, pool from eight animals for each). ***P < 0.05 vs. CD45^−^ from SNI-WT; ^##^*P* < 0.05 vs. CD11b^+^ from Sham-WT; ND, not detected. Data are presented as means ± S.E.M. Data were analyzed by two-way ANOVA followed by Bonferroni post-test **(B)** or by one-way ANOVA followed by Tukey post-test **(C–F)**.

Next, we sought to evaluate whether the antinociceptive effect of exogenous IL-27 on neuropathic pain would be dependent on IL-10. For this, WT and IL-10^−/−^ mice were submitted to peripheral nerve injury (SNI), and at day 10 after surgery we detected mechanical hypersensitivity development in these animals. Different groups of mice were treated with daily doses of recombinant IL-27 (100 ng/site) or vehicle up to day 14 (Figure 7A). As already shown, the treatment with recombinant IL-27 reduced the mechanical pain hypersensitivity in WT-SNI. However, this antinociceptive effect of IL-27 has not been observed in IL-10^−/−^-SNI mice (Figure 7B). Corroborating these data, the treatment of WT-SNI mice with recombinant IL-27 promoted an upregulation of *Il10* expression in the DRGs and spinal cord when compared to mice submitted to SNI treated with vehicle (Figures 7C,D). Supporting our previous finding that IL-27 receptor is mainly expressed in CD11b^+^ cells in the DRGs (Figure 3C), we found that the upregulatory effect of recombinant IL-27 treatment on IL-10 expression is only observed in CD11b^+^ cells compared to non-immune cells (CD45^−^ cells) (Figure 7E). Lastly, we sought to confirm that IL-10 cytokine expression after peripheral nerve injury depends on IL-27 action in myeloid cells in the DRGs. WT and IL-27^−/−^ mice were submitted to SNI or Sham surgeries. At day 10 after injury, DRGs were collected, and CD45^−^ (non-immune cells) and CD11b^+^ cells (macrophages) were isolated by FACS sorting. Then, we analyzed the gene expression of *Il10* in these types of cells. It was found that only CD11b^+^ cells from WT animals submitted to SNI expressed *Il10*, which was reduced in CD11b^+^ cells from IL-27^−/−^ SNI animals (Figure 7F). Altogether these results indicate that the antinociceptive effect of exogenous and endogenous IL-27 upon neuropathic pain caused by peripheral nerve injury depends on the induction of IL-10 expression on myeloid cells in the DRGs.

## Discussion

Neuropathic pain is a challenging condition often refractory to different therapies. It is well-known that neuroimmune–glia interactions play a crucial role in the development of neuropathic pain. In this context, understanding the function of each cytokine in neuropathic pain pathophysiology might provide potential opportunities to amplify the arsenal of treatment options available (52). The main signaling molecules of the immune system are cytokines, which can be largely categorized as either pro- or anti-inflammatory. Elevated pro-inflammatory cytokine signal has been associated with symptoms of pain after nerve injury, whereas cytokines such as IL-10 and IL-4 are associated with the downregulation of the immune system and neuropathic pain relief (47, 52–54). Here, we provided evidence that IL-27 counteracts neuropathic pain development through the induction of anti-inflammatory cytokine IL-10.

Our findings demonstrate that IL-27 protects animals against the development of mechanical and cold pain hypersensitivity after peripheral nerve injury, two major symptoms of neuropathic pain. On the other hand, our data indicate that IL-27 did not participate in the nociceptive pain caused by different stimuli modalities, which was different from a previous report showing that the deficiency of IL-27 and its signaling constitutively control thermal and mechanical nociceptive thresholds (19). Although the explanation for these differences is not immediately apparent, it could be due to the difference in the source of the mutant mice. Nevertheless, our data show that the expression of IL-27 receptor is very low in the nociceptive pathway at naïve condition, favoring the idea that IL-27 would have little or no participation in nociceptive pain. Despite this controversy, it was shown that IL-27-deficient mice present higher mechanical sensitivity when submitted to inflammatory and neuropathic pain models compared to WT animals (19), supporting our data. Furthermore, the data showing that peripheral nerve injury induced upregulation of IL-27 subunits (EBI3 and p28) and its receptor subunit (WSX-1) in important structures of the nociceptive pathway (DRGs and spinal cord) argue in favor of the regulatory role of IL-27 in the development of neuropathic pain.

The neuroimmune–glia interactions across the pain pathway play a crucial role in the development of neuropathic pain (4). For instance, peripheral nerve injury triggers the activation/proliferation of macrophages in the DRGs and glial cells (microglia and astrocytes) in the spinal cord (4). These glial cells mediate the development of neuropathic pain through the production of several neuroactive molecules (55–58). It is noteworthy that IL-27 receptor-specific subunit WXS-1 (17) is expressed in different leukocyte populations such as macrophages (18, 59, 60), microglia, and astrocytes from the brain of multiple sclerosis patients (21). Our results extend these findings, indicating that after peripheral nerve injury, WSX-1 expression in the DRGs and spinal cord was mainly observed in immune or glial cells. Based on those findings and on the fact that IL-27 is an important immunoregulatory cytokine (24, 28), we analyzed whether IL-27 counteracts neuropathic pain development through the modulation of macrophage/glial cell (microglia and astrocytes) activation in the DRGs and spinal cord, respectively. However, no significant changes in the expression of macrophage/glial cell activation markers and their derived pro-nociceptive cytokines (TNF and IL-1β) were detected after peripheral nerve injury in the DRGs and spinal cord from IL-27 null mice compared to WT mice. Thus, although IL-27 receptor seems to be confined in macrophage/microglia and astrocyte after peripheral nerve injury, IL-27 effect on neuropathic pain process appears to be independent on the regulation of these cells' activation/proliferation. These data corroborate with the previous study showing no role for IL-27 signaling in spinal cord microgliosis after peripheral nerve injury (19).

Concomitantly to the production of pro-inflammatory molecules by immune and glial cells at the sensory ganglia and spinal cord after peripheral nerve injury, there is also evidence suggesting the production of anti-inflammatory/anti-nociceptive molecules (12, 13, 50). Among these molecules, IL-10 seems to be one of the most important (15, 61, 62). The antinociceptive role of IL-10 has been extensively explored in different models of pathological pain (63–66). We found that the regulatory role of IL-27 in the development of neuropathic pain is dependent on IL-10 production in the DRGs and spinal cord. At least in the DRGs, IL-27 triggers the induction of IL-10 in macrophages. It is important to point out that IL-10 upregulation after peripheral nerve injury was not exclusively dependent on IL-27 since IL-10 production was not abrogated in IL-27 null mice. Thus, other direct or indirect mechanisms might be involved in the induction of IL-10, such as A2A and A3 adenosine receptor activation (67, 68). In addition, our data corroborate with a recent finding showing that the antinociceptive effect of IL-10 is not dependent on inhibition of neuroinflammation (69). Therefore, it is plausible to suggest that IL-10 might reduce neuropathic pain by acting directly on sensitive neurons. In fact, IL-10R1 is expressed in neurons, and its activation directly regulates the functions of the primary sensory neurons (70, 71).

The use of exogenous IL-27 has been recognized as a novel biological tool to treat a vast range of inflammatory and autoimmune disease (28, 30, 31, 72, 73). In addition to the endogenous involvement of IL-27 in counteracting peripheral nerve injury-induced neuropathic pain development, exogenously administered recombinant IL-27 was also able to reduce neuropathic pain. It is noteworthy that the effective dose of recombinant IL-27 did not alter the mechanical pain threshold, in contrast to previous findings (19). These might be explained by the different dose or route (intrathecal vs. systemic) of IL-27 administration. In addition, the dose of recombinant IL-27 used did not cause any sedative/motor impairment. Then, these data might indicate that IL-27 could be a useful tool to prevent/reduce neuropathic pain in clinical settings.

In summary, our study unraveled the role of IL-27 as a regulatory cytokine that counteracts the development of neuropathic pain through the induction of anti-nociceptive cytokine IL-10. In conclusion, these data provide new insights into the neuroimmune–glia interaction mechanisms involved in the development of neuropathic pain. Finally, they indicate that immunotherapies based on IL-27 could emerge as possible therapeutic approaches for the prevention of neuropathic pain development after peripheral nerve injury.

## Data Availability Statement

The datasets generated for this study are available on request to the corresponding author.

## Ethics Statement

Animal care and handling procedures were in accordance with the International Association for the Study of Pain guidelines (74) for those animals used in pain research, and they were approved by the Committee for Ethics in Animal Research of Ribeirão Preto Medical School -USP (Process no 211/2014).

## Author Contributions

MF designed and performed most of the experiments, analyzed the data, and wrote the manuscript. MD-F and FS-C designed and assisted in performing the experiments. RG, RK, FO, and DF assisted in performing some experiments. FC and JA-F reviewed the manuscript and provided expert discussion of the project. TC conceived the study, supervised the overall project, and wrote the manuscript. All authors contributed to manuscript revision and read and approved the submitted version.

### Conflict of Interest

The authors declare that the research was conducted in the absence of any commercial or financial relationships that could be construed as a potential conflict of interest.

## Supplementary Material

Figure S1 Representative gating strategies for flow cytometry analysis. After the specified days, DRGs were collected from mice, and CD45^−^ (non-immune cells) and macrophages (CD11b^+^Ly6G^−^) were isolated using FACS sorting. FSC-H/FSC-A preliminary gate was performed for all cytometry analysis to exclude cell debris and cell doublets. Next, CD45^+^ cells were gated on two populations, CD11b^+^Ly6G^+^ and CD11b^+^Ly6G^−^. Then, CD45^−^ and CD45^+^C11b^+^Ly6G^−^ were sorted. For all of the experiments, the sample purity was ~70% for CD11b^+^Ly6G^−^ cells and ~90% for CD45^−^ cells. We use ~25,000 cells to isolate mRNA in each group of experiment.

## Abbreviations

CNS: central nervous system
CX3CR1: motif chemokine receptor 1
DRG: dorsal root ganglion
EAE: experimental autoimmune encephalomyelitis
EBI3: epstein–barr virus-induced 3
ELISA: enzyme-linked immunosorbent assay
GFAP: glial fibrillary acidic protein
i.p: intraperitoneal injection
i.t: intrathecally injection
IBA-1 (*Aif1*): ionized calcium-binding adapter molecule 1
IL-4: interleukin 4
IL-10: interleukin 10
IL-13: interleukin 13
IL-17: interleukin 17
IL-18: interleukin 18
IL-1β: interleukin 1 beta
IL-27: interleukin 27
IL-27r: recombinant mouse IL-27 protein
mg: milligram
PBS: phosphate-buffered saline
PFA: paraformaldehyde
pg: picogram
Sham: false-operated animals
SNI: spared nerve injury
TGF-β: transforming growth factor beta
Th17: T helper 17 cell
TNFα: tumor necrosis factor alpha
Tr1: type 1 regulatory cells
WT: wild type.
