# Supplementary figures and images for: IL-27 Counteracts Neuropathic Pain Development Through Induction of IL-10
Source: Front Immunol. 2020 Jan 28;10:3059. doi: 10.3389/fimmu.2019.03059 (PMC6997342; doi:10.3389/fimmu.2019.03059)

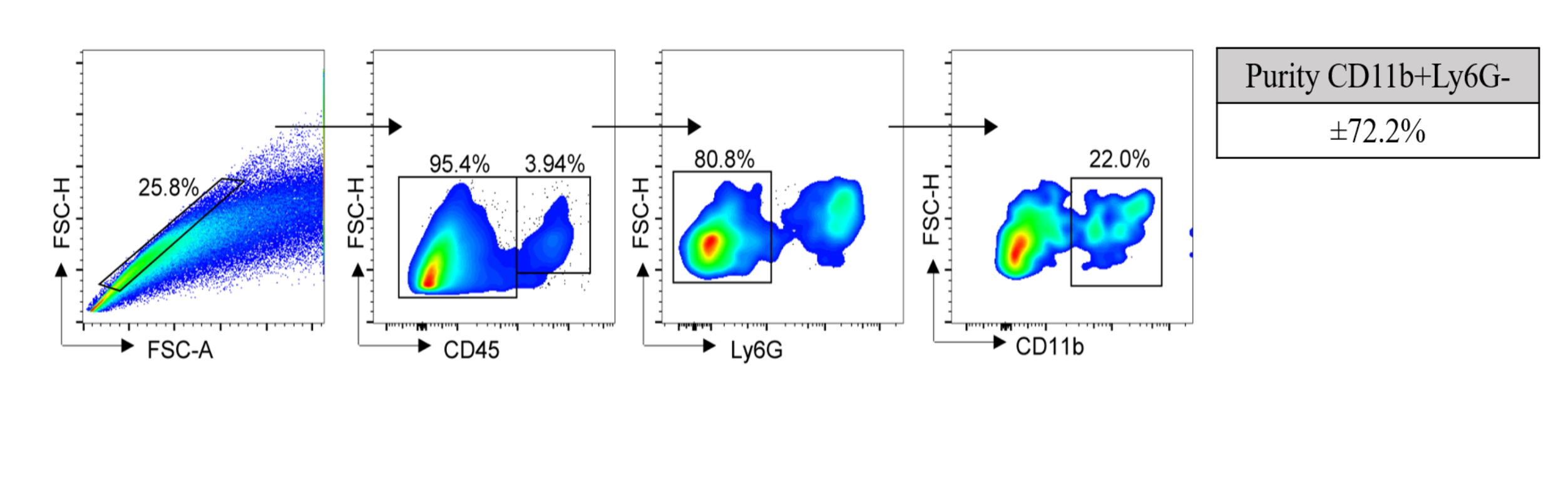

Supplement: Figure S1 — Representative gating strategies for flow cytometry analysis. After the specified days, DRGs were collected from mice, and CD45− (non-immune cells) and macrophages (CD11b+Ly6G−) were isolated using FACS sorting. FSC-H/FSC-A preliminary gate was performed for all cytometry analysis to exclude cell debris and cell doublets. Next, CD45+ cells were gated on two populations, CD11b+Ly6G+ and CD11b+Ly6G−. Then, CD45− and CD45+C11b+Ly6G− were sorted. For all of the experiments, the sample purity was ~70% for CD11b+Ly6G− cells and ~90% for CD45− cells. We use ~25,000 cells to isolate mRNA in each group of experiment. [file Image_1.TIF]
